# The Interplay Between Language, Executive Functioning, Theory of Mind and Socio‐Emotional Functioning – A Comparison Between Adolescents With and Without DLD

**DOI:** 10.1111/1460-6984.70168

**Published:** 2025-12-17

**Authors:** Elke Arts, Bram O. De Castro, Ellen Luteijn, Ben Elsendoorn, Constance T. W. M. Vissers

**Affiliations:** ^1^ Behavioural Science Institute Radboud University Nijmegen The Netherlands; ^2^ Research institute of Child Development and Education University of Amsterdam Amsterdam The Netherlands; ^3^ Royal Kentalis Utrecht The Netherlands; ^4^ Secondary School for Special Education for Children and Adolescents with Language and Communication Problems Royal Kentalis Arnhem The Netherlands

**Keywords:** adolescents, developmental language disorders, executive functioning, language, socio‐emotional functioning, theory of mind

## Abstract

**Background:**

Adolescents with developmental language disorder (DLD) are known to be at increased risk for socio‐emotional difficulties. Yet, there remains considerable uncertainty about how problems in language abilities, executive functioning (EF), and theory of mind (ToM) contribute to the socio‐emotional difficulties observed in this group. In addition, only a limited number of studies have compared adolescents with and without DLD on these underlying cognitive and linguistic domains.

**Aims:**

This study examined (1) differences between adolescents with and without DLD in language, EF, ToM, and socio‐emotional functioning, (2) associations among these domains, and (3) the unique contributions of language, EF, and ToM to socio‐emotional functioning.

**Methods and Procedures:**

Forty adolescents with developmental language disorder (DLD) and 36 typically developing (TD) peers, matched for age and education level, completed measures of receptive vocabulary, visuospatial working memory (Corsi Block‐Tapping Task), cognitive flexibility (Berg Card‐Sorting Test), cognitive ToM (ToMotion task), and affective ToM (Emotion Recognition Task). Parents completed the IKAN questionnaire, which served as the measure of socio‐emotional functioning. Group differences were assessed using independent‐samples *t*‐tests and Mann–Whitney *U* tests. Associations were examined using Spearman correlations. Multiple regression analyses were conducted with the IKAN total score as the outcome variable.

**Outcomes and Results:**

Adolescents with DLD scored significantly lower than their TD peers on receptive language, cognitive ToM, and socio‐emotional functioning as measured by the IKAN. They showed significantly reduced scores on seven of the eight socio‐emotional subscales. No significant group differences were found for visuospatial working memory, cognitive flexibility, or affective ToM. Receptive vocabulary correlated positively with cognitive flexibility, both ToM measures, and several IKAN subscales. Cognitive ToM showed consistent moderate associations with overall socio‐emotional functioning. In the regression model, cognitive ToM was the only significant unique predictor of socio‐emotional functioning (*R* ^2^ = 0.23).

**Conclusions and Implications:**

This study demonstrates that adolescents with DLD experience more difficulties in receptive language, cognitive ToM, and socio‐emotional functioning compared to their TD peers. Moreover, cognitive ToM appears to be a key predictor of socio‐emotional functioning across adolescents with and without DLD. Clinical and educational services should therefore extend support beyond language remediation and consider targeted interventions that strengthen cognitive ToM to improve socio‐emotional adjustment.

**WHAT THIS PAPER ADDS:**

*What is already known on this subject*

- Adolescents with developmental language disorders (DLDs) experience significant socio‐emotional difficulties, including higher levels of anxiety, depression, and peer victimization. Research highlights the role of cognitive factors, such as Theory of Mind (ToM) and executive functioning (EF), in socio‐emotional development, but studies specifically focusing on these constructs in DLD are limited. Language difficulties alone do not fully explain the socio‐emotional challenges, indicating that underlying cognitive deficits might play a key role.

*What this study adds to the existing knowledge*

- This study examines the relationship between language abilities, ToM, EF, and socio‐emotional functioning in adolescents with DLD. It explores how these cognitive factors uniquely contribute to socio‐emotional problems, providing a deeper understanding of the mechanisms at play in this population.

*What are the potential or actual clinical implications of this work?*

- This study highlights the need for individualized interventions for adolescents with DLD, given the significant variability in cognitive profiles and socio‐emotional functioning within this group. The findings suggest that cognitive factors, particularly ToM, may play a crucial role in predicting socio‐emotional difficulties, emphasizing the importance of targeting these areas in therapeutic interventions. Additionally, the lack of significant correlations between receptive language and socio‐emotional functioning suggests that both receptive and expressive language should be assessed to develop more comprehensive interventions aimed at improving social skills and emotional regulation.

## 1 Introduction

Adolescents with developmental language disorders (DLDs) experience problems in the understanding and expression of language, without any known underlying neurobiological cause (Bishop et al. 2017). DLD is one of the most prevalent neurodevelopmental disorders, with an estimated prevalence of 7% (Norbury et al. 2016). However, in terms of research, DLD remains underrepresented compared to other neurodevelopmental conditions (Kulkarni et al. 2022). Moreover, existing research on DLD primarily focuses on the expressive and receptive language problems and rarely on socio‐emotional functioning (Arts et al. 2022). This is disturbing because individuals with DLD do not only encounter significant language difficulties but are also at risk of socio‐emotional problems (Conti‐Ramsden et al. 2013; Fujiki et al. 2002). Recent findings indicate that the social–emotional and behavioural difficulties observed in children with DLD are comparable in severity to those seen in autism spectrum disorder (ASD) and attention deficit hyperactivity disorder (ADHD) (Löytömäki et al. 2023). Adolescents with DLD reported, for example, higher levels of anxiety, depression and victimization in combination with higher emotional problems and difficulties in peer relationships compared to their typically developing (TD) peers (Conti‐Ramsden and Botting 2008; Durkin and Conti‐Ramsden 2007). In addition, adolescents with DLD reported reduced self‐confidence in social situations and higher levels of social stress (Wadman et al. 2011). Despite these associated problems, the neurocognitive aspects underlying socio‐emotional functioning in adolescents with DLD are not yet clearly understood (Smit et al. 2019). There are indications that difficulty with language production and comprehension, however, do not completely explain the socio‐emotional problems in this clinical group (Fujiki et al. 2004). Understanding underlying cognitive abilities in socio‐emotional functioning appears to be especially crucial for individuals who are already vulnerable, such as those with DLD, due to their communication and language difficulties.

### 1.1 Socio‐Emotional Functioning in Adolescents With DLD

Socio‐emotional functioning is described as multiple processes and abilities that enable individuals to communicate successfully and manage emotions effectively in social contexts (Damon et al. 2006). Research shows that the frequently reported socio‐emotional problems in children with DLD arise at an early age. Preschoolers with language impairments showed, for example, significantly more difficulties in social skills than preschoolers with TD language skills (Stanton‐chapman et al. 2007). Moreover, these preschoolers exhibit specific difficulties in assertiveness, peer social skills, and frustration tolerance, and were more likely to be dependent and isolated in the classroom (McCabe 2005). In addition, parents of school‐aged children with DLD reported significantly lower scores on self‐control, and teacher ratings showed significantly lower scores on assertiveness in comparison with their TD peers (McCabe and Meller 2004). These problems in socio‐emotional functioning tend to intensify towards adolescence. Moreover, many children and adolescents with DLD experience limited peer relationships and reduced social contact outside the home (Löytömäki et al. 2023). This limited social participation puts them at risk of loneliness and social isolation, which may further reduce opportunities to practice and refine socio‐emotional skills. Over time, this can create a self‐reinforcing cycle in which loneliness, withdrawal, and emotional misunderstanding mutually reinforce each other. Longitudinal data support this pattern, showing a steady increase in peer‐related difficulties as children with DLD grow older. For example, 28% of children with DLD experience difficulties in social interactions with peers at the age of 8; this percentage rises to 33% by the age of 11 and is reported to reach 39% among adolescents by the age of 16, according to a longitudinal follow‐up of the same cohort (*n* = 234 at age 7; *n* = 203 at age 8; *n* = 167 at age 11; *n* = 103 at age 16) conducted by St Clair et al. (2011). This increase in socio‐emotional problems during adolescence is likely related to the fact that the role of parents gradually diminishes, while friendships, peer contact, and social interactions become increasingly important for social and emotional development (Larson and Richards 1991). This transition requires adolescents to engage in complex social interactions and develop a broad network of relationships with their peers (Lamblin et al. 2016). However, their primary language problems, combined with reduced and/or negative social experiences in their past, make it difficult for adolescents with DLD to develop the self‐confidence needed to engage in successful social interactions and form new social connections during adolescence (Wadman et al. 2008). This is particularly disturbing, as it may contribute to a vicious cycle in which individuals with few or negative relationships are more likely to experience states of loneliness and depression. Additionally, a chronic history of victimization and rejection is similarly associated with increased levels of depression and social anxiety (Foulkes and Blakemore 2018). Such emotional distress, in turn, hinders the formation of social relationships, with social isolation or peer rejection potentially leading to psychological states that resemble physical pain (Kross et al. 2011). More generally, early life deficits in socio‐emotional functioning have been investigated as antecedents of later subclinical behavioural and psychiatric problems (Briggs‐gowan and Carter 2008). Thus, given the high prevalence and potential severe consequences of social‐emotional difficulties in youth with DLD, it seems particularly important to understand these socio‐emotional challenges and to develop adequate methods to stimulate adequate socio‐emotional functioning in this population.

### 1.2 The Role of ToM and EF in Socio‐Emotional Functioning

According to a neuropsychological perspective, problems in socio‐emotional functioning derive from a complex interplay between difficulties in language and underlying cognitive deficits in mentalization (Theory of Mind [ToM]) and self‐regulation (executive functioning [EF]) (Tomas and Vissers 2019). ToM involves the ability to understand and interpret one's own and others’ emotions (affective ToM), perspectives and desires (cognitive ToM) (Wellman 2018). EF can be defined as higher cognitive control processes that regulate human cognition and behavior in complex and novel situations (Diamond 2013). Within the domain of EF, inhibitory control, working memory and cognitive flexibility are considered as the three core functions (Miyake et al. 2000). More specifically, inhibition refers to the ability to suppress irrelevant or unwanted information, impulses, and thoughts, and inappropriate or awkward behaviours (Rhoades et al. 2009). Working memory is defined as the set of processes that enable an individual to temporarily store and manipulate information (Barkley 1997). Cognitive flexibility involves the ability to adapt thoughts and behaviours to changing demands, perspectives, or environments (Chevalier and Blaye 2009).

Over the years, various studies have been conducted into the role of cognitive factors in the development of socio‐emotional functioning. Meta‐analyses showed that ToM and EF play major roles in the development of socio‐emotional functioning in non‐clinical populations (for cognitive ToM, see Slaughter et al. 2015; for affective ToM, see Zhang et al. 2023; for EF, see Schoemaker et al. 2013). A meta‐analysis, for example, found that TD children with higher cognitive ToM scores were more popular (Slaughter et al. 2015). Comparatively, poorly developed ToM abilities predicted being victimized and bullied in adolescents (Shakoor et al. 2012). In addition, weaker emotion recognition skills (affective ToM) were associated with higher internalizing problems, such as anxiety and depression (Zhang et al. 2023). Similarly, EF impairments, particularly in working memory, were related to peer rejection, poor overall social competence and impaired conflict resolution skills in school‐aged children (Mcquade et al. 2013). Moreover, difficulties in inhibition were associated with externalizing behaviour problems (Schoemaker et al. 2013).

### 1.3 The Role of Language in EF and ToM

The development of ToM and EF starts at birth and seems to be completely intertwined with the development of language (Camminga et al. 2021). The reason to assume this entangled relation is that language as well as ToM and EF are considered to develop in a hierarchical manner. Early stages of ToM, like joint attention and imitation, start developing around the age of 9 months, progressing towards more advanced stages such as emotion recognition and false belief understanding (Vissers and Koolen 2016). In terms of EF, children initially seem to develop relatively simple forms of EF, such as behavioural inhibition, before developing to more mature forms of EF (e.g., planning) (Vissers et al. 2015).

This entangled relationship between language and cognitive abilities is also demonstrated by several studies in non‐clinical populations. For example, one study showed that maternal mental state talk appears to be related to children's later ToM abilities (Taumoepeau and Ruffman 2008). Another study found that syntax and semantics contribute positively to more advanced ToM stages, such as false belief reasoning (Slade and Ruffman 2005). In addition, a meta‐analysis found a moderate correlation (*r* = 0.43) between children's linguistic abilities and false‐belief task performance (Milligan et al. 2007). It should be noted, however, that many ToM measures—particularly false‐belief tasks—are linguistically demanding and therefore partially reflect participants’ language comprehension skills. This interdependence may underlie part of the associations reported in previous studies. Moreover, ToM is best conceptualized as a continuum of interrelated cognitive, linguistic, and social abilities—ranging from early nonverbal social understanding to advanced perspective‐taking—rather than as a single discrete construct (Korkmaz 2011).

Furthermore, language skills at age 5–6 were shown to predict emotion recognition at age 10–12 (Griffiths et al. 2020). Studies in the field of EF have shown reciprocal associations between EF and language, with linguistic abilities emerging as the strongest predictor (Slot and von Suchodoletz 2018).

### 1.4 ToM and EF in DLD

Due to their parallel development, it is suggested that the cognitive impairments in DLD may be explained by the problems in the synthetization of language with precursors of EF and ToM (Camminga et al. 2021). Some evidence for this is provided by studies conducted on preschoolers with DLD who already show a reduced development of ToM and EF, in combination with language problems (for ToM, see Vissers and Koolen 2016; for EF, see Vissers et al. 2015). Subsequently, these problems in EF and ToM appear to persist in school‐aged children with DLD, as several meta‐analyses showed reliable differences between children with and without DLD. School‐aged children with DLD, for example, tend to have more problems with inhibitory control, cognitive flexibility and visuospatial working memory (Pauls and Archibald 2016; Vugs et al. 2013). Additionally, a meta‐analysis in the area of ToM showed that school‐aged children with DLD had substantially lower ToM performance in comparison with TD children (Nilsson and de López 2016). Children with DLD had, for example, significantly lower emotion recognition (Löytömäki et al. 2020) and emotion discrimination skills (Löytömäki et al. 2023) than controls. Unfortunately, less research has been done on the persistence of these cognitive problems into adolescence and adulthood. However, a study by Clegg et al. (2005) of 17 males with receptive DLD showed that these males still showed significant ToM difficulties into their mid‐thirties. Additionally, a study by Hughes et al. (2009), including 21 adolescents with DLD and 21 TD peers matched for age and gender, compared parent and self‐ratings of EF in adolescents with DLD and their TD peers. Results showed that 75% of the parents of the DLD group rated their adolescent's EF as being in the clinically impaired range, compared with 10% in the TD group. This significant difference was not found based on self‐ratings, as the adolescents with DLD rated themselves much more positively than their parents regarding their EF. Altogether, since EF and ToM have been suggested to be important underlying factors for the development of social‐emotional functioning, it is important to identify cognitive differences in these constructs between adolescents with and without DLD.

### 1.5 Socio‐Emotional Functioning and Cognitive Constructs in Adolescents With DLD

Only a limited number of studies have been conducted on the socio‐emotional problems and underlying cognitive constructs in adolescents with DLD (Arts et al. 2022; Smit et al. 2019). A recent study by Smit (2024) on EF and ToM in relation to social‐emotional problems in adolescents with DLD found higher problem scores on the subscale ‘social interaction’, but no diminished socio‐emotional functioning on the subscales ‘attention’, ‘oppositional problems’ and ‘anxiety and mood problems’, in comparison with the norm tables of TD adolescents on the Social Emotional Questionnaire (Scholte and van der Ploeg 2017). Positive correlations were found between ToM and the subscale ‘interaction’. No correlations were found between EF and social‐emotional functioning. Limitations of this study were that no language measures were included. As a result, no conclusions could be drawn regarding the extent to which language abilities influence cognitive and socio‐emotional problems.

### 1.6 The Present Study

Developing effective support for youth with DLD requires a thorough understanding of factors underlying their socio‐emotional problems. To this higher end, this study aimed to investigate the relationship between language, cognitive abilities (EF, ToM), and socio‐emotional functioning in adolescents with and without DLD. The first goal of this study was to replicate the few earlier studies showing differences in language, cognitive abilities (EF and ToM), and socio‐emotional functioning between a group of adolescents identified with DLD and their TD peers. The second aim of this study was to investigate the relationships between language, cognitive abilities (ToM/EF) and socio‐emotional functioning. Based on the current literature, we expected to see significant associations between linguistic abilities, EF and ToM. In addition, we expected that all three variables – language, EF and ToM – would be associated with socio‐emotional functioning. The third goal of the study was to determine the extent to which language and cognitive abilities (ToM/EF) could each uniquely contribute to explaining variance in socio‐emotional functioning. Based on the neuropsychological model, we hypothesize that linguistic abilities, EF and ToM all predict variance in socio‐emotional functioning.

## 2 Method

### 2.1 Sample

Participants of the DLD group were 40 adolescents (25 boys, 15 girls), aged 13–17 years old, *M* = 14.58, SD = 1.23. Inclusion criteria were having a DLD diagnosis and age between 12 and 17 years. The participants of the DLD group were recruited from three secondary schools for special education of children with communication problems in the Netherlands. The control group consisted of 36 adolescents (20 boys, 16 girls), who could be considered TD peers, aged 12–16 years old, *M* = 13.98, SD = 1.38. Controls were recruited from 7 regular secondary schools in the Netherlands. All participants attended the same educational level (VMBO; pre‐vocational secondary education).

Informed consent was obtained from the participants and their parents/caregivers. Participants and their parents/caregivers received a gift voucher of 15 euros as a reward for participation. The study was approved by the Ethics Committee of the Faculty of Social Sciences of the Radboud University (ECSW‐2022‐065) on September 16, 2022.

### 2.2 Diagnosis

The participants in the DLD group all attended special education for students with communication problems. In accordance with the admission requirements for this type of education (Siméa 2017), all participants had a DLD diagnosis established by a multidisciplinary team at an audiological centre or speech‐and‐language therapist according to the DLD guideline (NVLF 2017). To receive a diagnosis of DLD, participants must meet the following criteria: (1) no severe hearing problems, as assessed by an audiologist; (2) typical non‐verbal intelligence, as assessed by a psychologist; (3) no neurological problems; and (4) severe and persistent language problems affecting communicative effectiveness (SIAC 2023).

To assess the severity of the language problems, a speech‐language therapist at the audiological centre administered several standardized tests: the Clinical Evaluation of Language Fundamentals (CELF‐5; Wiig et al. 2019), the Schlichting Test for Language Comprehension (Schlichting et al. 2010a), the Schlichting Test for Language Production (Schlichting et al. 2010b), and the Peabody Picture Vocabulary Test (PPVT; Schlichting 2005). A diagnosis of DLD was considered only when individuals met one of the following criteria: (1) a score of 2 or more standard deviations below the average in one of the four language domains (i.e., speech, grammar, semantics, pragmatics), (2) a score of 1.5 or more standard deviations below the average in two domains, or (3) a score of 1 or more standard deviations below the average in three domains. These deviations had to be confirmed by results from at least two different language assessments.

### 2.3 Experimental Tests

The task battery included six tasks. These tasks were designed to assess the level of language, EF, ToM and socio‐emotional functioning in the participants.

#### 2.3.1 Language

##### 2.3.1.1 Receptive Vocabulary

Receptive vocabulary was assessed using the Dutch version of the Peabody Picture Vocabulary Task‐III‐NL (PPVT‐III‐NL, BRON). The PPVT is a widely employed, norm‐referenced assessment tool for word comprehension in individuals aged 2 to 90 years.

The questions in the PPVT are in multiple‐choice format. The individual is required to correctly identify which of the four presented pictures represents the spoken word. The participant starts with the block corresponding to his/her age. A block consists of 12 words. If the participant makes fewer than 5 mistakes in the starting block, the next block is administered. If the participant makes 5 or more mistakes, an easier block is administered. The test stops when the participant has made 8 or more mistakes in one block. The total score is calculated by subtracting the number of mistakes from the break item. Additionally, age‐corrected scores were calculated (WBQ). These age‐corrected scores were then analysed.

#### 2.3.2 EF

##### 2.3.2.1 Visual working memory

We administered the Corsi Block‐Tapping Task (Corsi 1973) to measure visuospatial working memory. Participants completed this task using the free and computerized version of the Psychology Experiment Building Language (PEBL; Mueller and Piper 2014) software (version 0.14).

**FIGURE 1:**
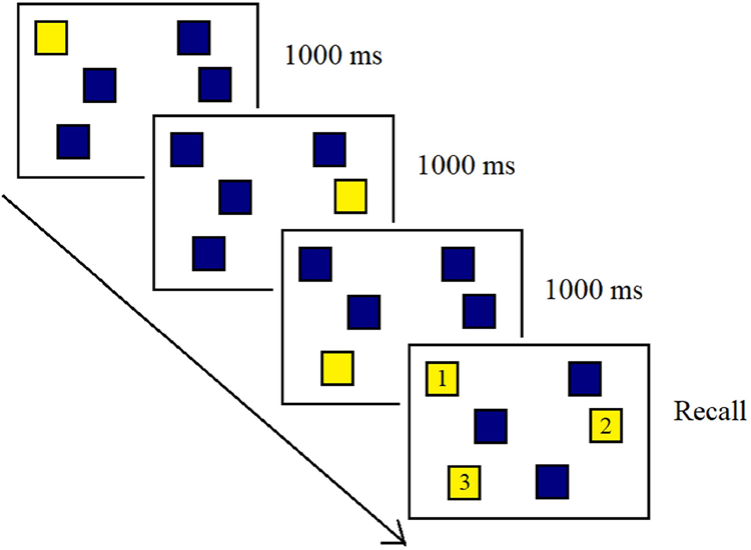
Schematic representation of the Corsi Block‐Tapping Task showing the forward recall condition.

At the start of the task, participants were shown a 3 × 3 array of nine blue blocks presented on a black background. On each trial, a subset of these blocks briefly changed colour (from blue to yellow) in a specific sequence (see Figure 1). Each block remained highlighted for 1000 ms, followed by a 250 ms inter‐stimulus interval before the next block in the sequence lit up.

Participants were instructed to observe the sequence carefully and then reproduce the exact same order by tapping the corresponding blocks by clicking with a mouse. The initial sequence length consisted of two blocks, and sequence length increased by one after each successfully reproduced pair of sequences. Each sequence length was presented twice, in two separate trials. If the participant failed to reproduce two consecutive sequences of the same length correctly, the task automatically terminated. The total number of possible sequence lengths ranged from 2 to 9 blocks, resulting in a maximum of 16 trials in total. The entire test took approximately 5 to 7 min to complete, depending on the participant's performance. The total score is calculated by multiplying the number of correct sequences by the maximum number of blocks correctly repeated, providing a more sensitive and reliable index of visuo‐spatial working memory than the traditional span score (Kessels et al. 2000).

##### 2.3.2.2 Cognitive Flexibility

The Berg Card‐Sorting Test (BCST; Berg 1948) is administered to assess cognitive flexibility. Participants completed this task using the PEBL's BCST, which is a free and computerized version of the original test (Mueller and Piper 2014) (version 0.14).

**FIGURE 2:**
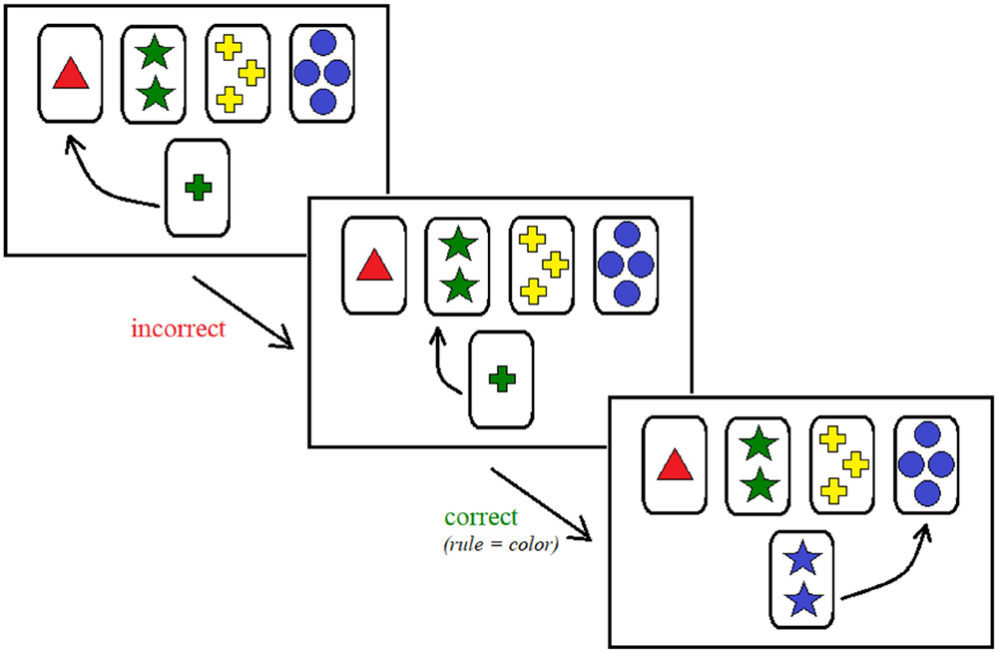
Schematic representation of the Berg Card‐Sorting Test.

During the task, participants were presented with four key cards displayed horizontally at the top of the screen. Each key card differed from the others in three possible dimensions: colour (red, green, blue, yellow), shape (triangle, star, cross, circle), and number of symbols (one to four). A response card appeared one at a time at the bottom of the screen, and participants were instructed to match the response card to one of the four key cards by clicking on the card they believed was correct (see Figure 2). The correct matching rule (colour, shape, or number) was not disclosed to the participant and had to be inferred from feedback. After each response, participants received immediate feedback on the screen indicating whether the response was ‘correct’ or ‘incorrect.’ Based on this feedback, participants had to deduce the current sorting principle.

A sorting rule was considered successfully acquired after 10 consecutive correct matches. Upon reaching this criterion, the sorting rule automatically changed to one of the two remaining dimensions without prior notice. Participants were required to adapt their sorting strategy accordingly. The task continued until eight rule shifts were completed or until the participant reached the maximum of 128 trials. Each response card remained on screen until a response was made, and the feedback display lasted 1000 ms before the next trial began. The total testing time typically ranged between 8 and 12 min, depending on performance and processing speed. The conceptual level responses (i.e., the number of three or more correct trials which occurred consecutively) were used for analysis.

#### 2.3.3 ToM

##### 2.3.3.1 Cognitive ToM

**FIGURE 3:**
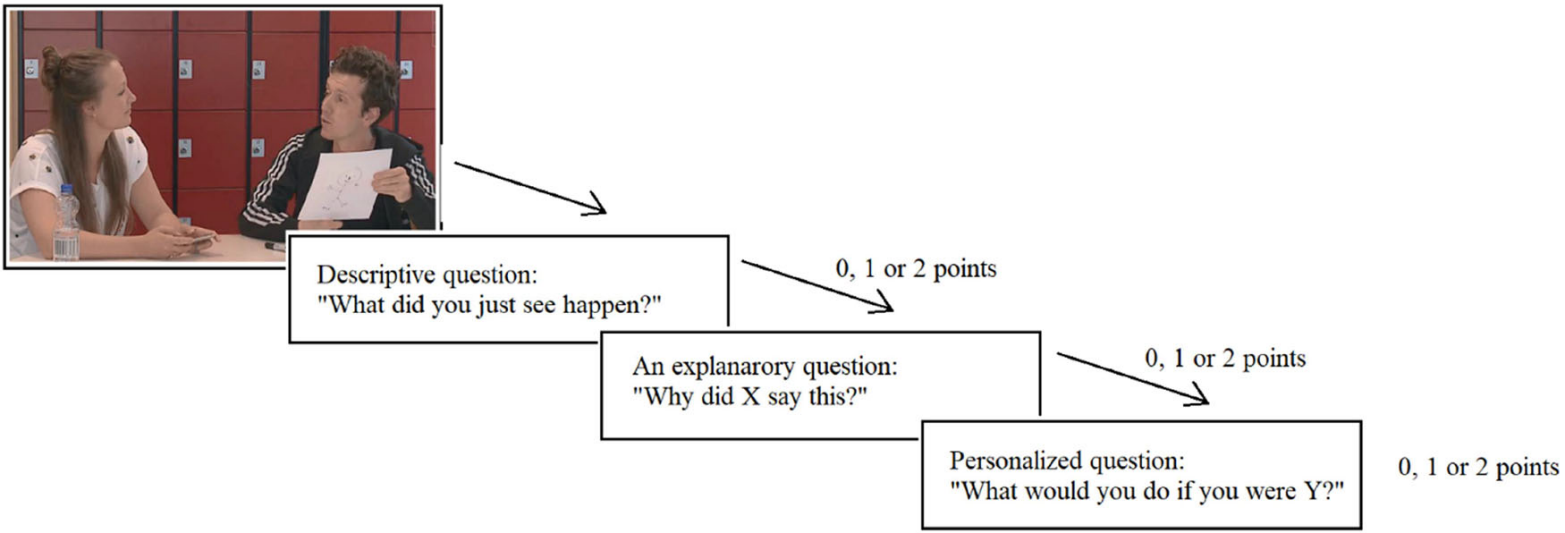
Schematic representation of the ToMotion Task (Cognitive ToM).

Cognitive ToM abilities were measured with the ToMotion task (Smit et al. 2022), a newly developed and empirically validated instrument designed to measure cognitive ToM in adolescents with language and communication problems. The task has shown good construct validity and internal reliability (*α* = 0.76), although it is not yet standardized. The task consists of 12 Dutch‐spoken videoclips displaying everyday‐life social interactions (i.e., joke, sarcasm, lie) between two peers. Based on these videoclips, the participant is asked to take the perspective of the peers and formulate their own response regarding the situation after each clip by answering three questions: (1) a descriptive question, (2) an explanatory question, and (3) a personalized question (see Figure 3). These questions evaluate whether the participant is able to recognize social cues, understand the intentions of peers, and respond appropriately based on those intentions. The responses given were assessed with 0, 1, or 2 points according to the example answers provided in the manual. Item scores were summed into a total score, where a maximum of 72 points could be achieved.

##### 2.3.3.2 Affective ToM

**FIGURE 4:**
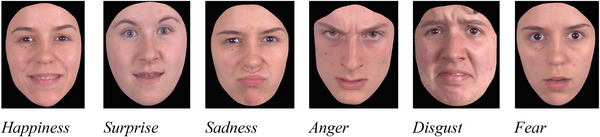
Schematic representation of the six basic emotion expressions of the ERT (Affective ToM).

**FIGURE 5:**
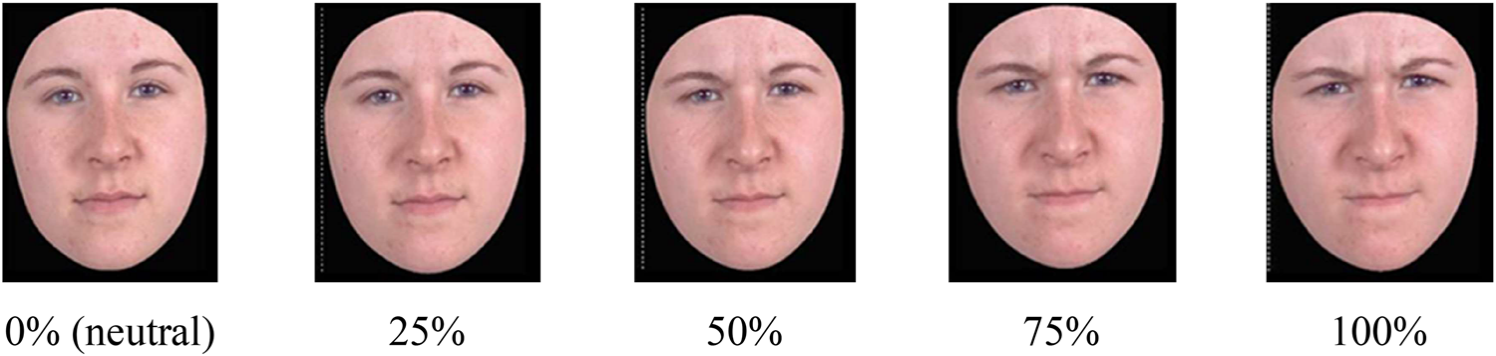
Schematic representation of the Emotion Recognition Task (ERT) displaying the different expression intensity levels (0%, 25%, 50%, 75%, and 100%) for the anger condition.

Affective ToM was measured with a Dutch translation of the Emotion Recognition Task [ERT] (Montagne et al. 2007). This test is a computerized task for identifying emotions. During the test participants were asked to watch a dynamic image of a face that slowly changed into a facial expression (such as happiness, surprise, sadness, anger, disgust and fear) (see Figure 4). The task consists of 96 facial expression morphs, which were presented as animations that gradually changed from a neutral expression (0%) to one of four intensity levels (25%, 50%, 75%, or 100%) (see Figure 5). Correct responses were scored as 1, and incorrect responses as 0, and a total correct score was used for analysis.

#### 2.3.4 Socio‐Emotional Functioning

Socio‐emotional functioning is measured with the IKAN‐questionnaire [Dutch: ‘IK‐en‐de‐ANder‐lijst’ = Me and Others list] (Van Hoof et al. 2023), filled in by parent(s). The test consists of 34 items, divided into 8 categories (i.e., task approach, dealing with difficulties, standing up for yourself, self‐knowledge, dealing with feelings and behaviour, adapting to people and environment, working together and presenting yourself positively to others). Items could be answered on a 4‐point Likert scale (1 = *My child can't do it yet*, 2 = *My child can do it with an adult's help*, 3 = *My child can do it independently with a tool*, 4 = *My child can do it completely independently*). A score of ‘3’ is considered the functional ‘standard’. This standard reflects a reasoned criterion rather than a statistically derived norm, indicating sufficient independence but possibly with minimal support (e.g., the use of visual cues or structured guidance). According to the test developers, this level corresponds to the average level that students with communication or language disorders at Kentalis schools achieve after completing their four‐year education. Both total scores and scores per subcategory were analysed.

In the current sample, the internal consistency of the subscales was acceptable to good, with Cronbach's *α* values ranging from 0.73 to 0.86: Task Approach (*α* = 0.76), Difficulties (*α* = 0.73), Standing Up (*α* = 0.86), Self‐Knowledge (*α* = 0.75), Dealing (*α* = 0.86), Adapting (*α* = 0.75), Working Together (*α* = 0.85), and Presenting (*α* = 0.79).

### 2.4 Procedure

Testing took place in a small and quiet room at the participants’ school. Participants were tested individually, and testing took up to 90 min. Participants completed the experimental tasks in a single session. Task order was predetermined (i.e., CORSI, ToMotion, BCST, PPVT, ERT, IKAN). Task instructions were read out loud to the participants in both groups because of the language difficulties in the DLD group. Additionally, participants were given a practice item for all tasks. The computerized (cognitive) tests were presented on an HP EliteBook 845 G7 (14 in., 60 Hz monitor). The PPVT was presented on paper; participants were asked to point to the correct answer. To avoid testing fatigue, a mandatory 10‐min break was scheduled after completion of 3 tests. Parents filled out the digital questionnaire about the social‐emotional functioning of their children via a personal link in their email. After completion the answers were sent directly to the researcher.

### 2.5 Analyses

SPSS for Windows (version 27) was used for statistical analyses. Independent sample *t*‐tests and Mann–Whitney *U* tests were used to test whether the linguistic, cognitive and socio‐emotional abilities in the DLD group would differ from the group of TD adolescents. Preliminary analyses were conducted to verify that the assumptions of normality were met. The normality of all dependent variables was examined using Shapiro–Wilk tests within each group. Several variables showed significant deviations from normality in both the DLD and TD groups (*p* < 0.05); therefore, non‐parametric Mann–Whitney *U* tests were applied for between‐group comparisons.

Correlations were calculated over the total sample (DLD and TD) to assess whether there were any relationships between linguistic, cognitive (ToM/EF) and socio‐emotional abilities. In addition, we examined the relationship between language, EF, ToM and the subscales of socio‐emotional functioning. Correlations between all variables were examined using Spearman correlations, since most variables were non‐normally distributed or contained outliers. We chose not to remove the outliers because they were not extreme outliers and therefore reflective of actual performance by the heterogeneous participant group. Missing data were deleted pairwise, meaning that participants were excluded only from analyses involving the variables for which their data were missing, while their available data were retained for all other analyses.

To examine the factors explaining variance in socio‐emotional functioning, multiple regression analysis was conducted. Informed by the neuropsychological model that attempts to explain the socio‐emotional problems in adolescents with DLD, we regressed socio‐emotional functioning on receptive language, visual working memory (EF), cognitive flexibility (EF), and cognitive and affective ToM simultaneously. In the regression analyses, the total IKAN score served as the dependent variable, representing overall socio‐emotional functioning. Separate regression models for individual subscales were not performed, as the subscales are each based on a small number of items, resulting in higher measurement error and lower reliability. Participant recruitment was part of a broader research project and was not directly based on an a priori power estimation. However, a formal power analysis was conducted for the multiple regression analyses, as these represented the most complex statistical model in the study. The analysis confirmed that the available sample size (*N* = 76) provided sufficient statistical power (1 – *β* = 0.80, *α* = 0.05) to detect medium‐sized effects.

## 3 Results

Prior to the main analyses, statistical assumptions and missing data were examined. Of the 76 respondents, we had 1 missing on the ERT, and 3 missing on the IKAN. These were due to incorrectly saved data (ERT) and uncompleted questionnaires by parents (IKAN).

As the first main analysis, between‐group differences were tested with independent sample *t*‐tests and Mann–Whitney *U* tests. Results showed that the TD group significantly outperformed the DLD group on linguistic abilities, cognitive ToM and socio‐emotional functioning (see Table 2). No significant differences were found between the groups on EF and affective ToM results. Moreover, the DLD group showed larger variance on all variables compared to the TD group, as evidenced by the higher standard deviations in the DLD group (see Table 1).

**TABLE 1:** Comparisons between the DLD and TD group on language, EF, ToM and socio‐emotional functioning.

Concept	DLD (*N* = 40) Mean (SD)	TD (*N* = 36) Mean (SD)	DLD Min–Max	TD Min–Max	Test statistics	*p* values
Receptive vocabulary	81.70 (12.45)	94.78 (8.51)	57–99	83–116	*U* = 300.50	< 0.001
Visual working memory (EF)	50.80 (18.25)	50.11 (16.63)	4–104	20–84	*U* = 674.50	0.632
Cognitive flexibility (EF)	88.35 (14.31)	93.97 (7.28)	37–105	78–104	*U* = 534.50	0.053
Cognitive ToM	46.13 (6.58)	54.86 (4.54)	35–63	43–64	T (69.46) = −6.79	< 0.001
Affective ToM	52.10 (9.42)	53.60 (8.44)	29–69	25–68	*U* = 654.00	0.625
Socio‐emotional functioning	94.00 (17.74)	116.91 (10.99)	59–131	84–132	*U* = 196.50	< 0.001

*Note*: Affective ToM; *N* = 38 for TD. Socio‐emotional functioning; *N* = 35 for TD, *N* = 38 for DLD.

Moreover, these differences were also significant on seven of the subscales of the IKAN (see Table 3). Only the difference on subscale ‘adapting to people and environment’ was not significant. When we look specifically at the norm scores per subscale, it can be seen that the DLD group only meets the applied norm score (norm = 3) in the areas of ‘adapting to people and environment’ and ‘presenting yourself positively to others’. In comparison, the TD group meets the established norm on all scales. In addition, the DLD group again showed greater variability in scores on all subscales of social‐emotional functioning compared to the TD group, as evidenced by higher standard deviations in the DLD group (see Table 2).

**TABLE 2:** Comparisons between the DLD and TD group on the subscales of socio‐emotional functioning.

Subscales socio‐emotional functioning	DLD (*N* = 38) Mean (SD)	Normative mean (Norm = 3)	TD (*N* = 35) Mean (SD)	Normative mean (Norm = 3)	Test statistics	*p* values
Task approach	14.21 (2.88)	2.84	17.06 (2.33)	3.41	*U* = 298.00	< 0.001
Dealing with difficulties	7.47 (2.01)	2.49	9.34 (1.88)	3.11	*U* = 333.00	< 0.001
Standing up	11.18 (3.13)	2.80	14.89 (1.84)	3.72	*U* = 238.50	< 0.001
Self‐knowledge	7.63 (2.09)	2.54	10.26 (1.67)	3.42	*U* = 224.00	< 0.001
Feelings and behavior	18.66 (5.17)	2.67	24.20 (3.67)	3.46	*U* = 269.50	< 0.001
Adapting	10.55 (2.00)	3.52	11.43 (0.66)	3.81	*U* = 525.00	0.096
Working together	11.47 (3.25)	2.87	14.57 (1.77)	3.64	*U* = 296.50	< 0.001
Presenting yourself	12.82 (2.99)	3.20	15.17 (1.18)	3.79	*U* = 313.50	< 0.001

To investigate the possible relationships between linguistic and cognitive abilities and socio‐emotional functioning, two‐tailed correlations between all variables were calculated (see Table 3). Results showed that receptive language abilities were significantly and positively correlated to cognitive flexibility (BSCT), affective ToM (ERT), cognitive ToM (ToMotion) and the subscales ‘task approach’, ‘difficulties’, ‘adapting’, and ‘working together’ of socio‐emotional functioning (IKAN). In addition, a significant correlation was found between working memory (CORSI) and affective ToM (ERT). Furthermore, cognitive ToM (ToMotion) correlated significantly and positively with socio‐emotional functioning (IKAN).

**TABLE 3:** Spearman correlation analyses of the relationship between linguistic, cognitive and socio‐emotional abilities in both groups.

	Receptive vocabulary	Working memory	Cognitive flexibility	Cognitive ToM	Affective ToM
Receptive vocabulary	—				
Working memory	−0.05	—			
Cognitive flexibility	0.23^*^	0.07	—		
Cognitive ToM	0.46^**^	0.21	0.19	—	
Affective ToM	0.26^*^	0.26^*^	0.09	0.17	—
Socio‐emotional functioning (Total)	0.21	0.08	0.04	0.47^**^	0.02
‐ Task approach	0.24^*^	0.08	−0.02	0.41^**^	−0.13
‐ Dealing with difficulties	0.29^*^	0.12	0.11	0.48^**^	0.14
‐ Standing up	−0.03	0.18	0.04	0.34^**^	−0.01
‐ Self‐knowledge	0.22	−0.03	0.09	0.40^**^	0.01
‐ Feelings and behavior	0.09	0.09	0.03	0.36^**^	−0.00
‐ Adapting	0.23^*^	0.06	−0.07	0.28^*^	0.12
‐ Working together	0.25^*^	0.01	−0.08	0.40^**^	−0.02
‐ Presenting yourself	0.14	0.16	0.01	0.31^**^	0.03

*Note*: *N* = 72–76.

* Indicates that the correlation is significant at the 0.05 level.

** Indicates that the correlation is significant at the 0.01 level.

The final step in the data analyses was to regress socio‐emotional functioning on language, EF and ToM. A multiple regression analysis was used to assess the relative contribution of these variables. In order to identify influential data points, Cook's distance (*D*) was calculated for each observation. According to the commonly used threshold of *D* > 1 (Cook and Weisberg 1982), no observations were identified as potentially too influential. However, one observation (*D* = 0.33) had substantially higher influence than other observations. Because of this, we reran the model again without this influential case. The results of this analysis did not differ substantially from the original analysis, and there was no reason to doubt the validity of this case. For this reason, we report the regression analysis based on all cases here.

Results of the multiple regression analysis showed that the overall model was statistically significant, *F*(5, 66) = 3.88, *p* = 0.004, explaining 23% of the variance in socio‐emotional functioning (*R* ^2^ = 0.23, Adjusted *R* ^2^ = 0.17). Cognitive ToM emerged as the only significant predictor (see Table 4).

**TABLE 4:** Summary details of multiple regression predicting socio‐emotional functioning.

	Unstandardized *B*	Coefficients SE	*β*	Sig.
Constant	44.57	22.65		0.053
Receptive vocabulary	−0.22	0.21	−0.14	0.302
Working memory	−0.16	0.14	−0.15	0.251
Cognitive flexibility	0.14	0.19	0.09	0.451
Cognitive ToM	1.43	0.36	0.55	< 0.001
Affective ToM	0.05	0.24	0.02	0.841

## 4 Discussion

The overall aim of the current study was to investigate the interplay between socio‐emotional functioning, language, ToM and EF in adolescents with and without DLD. Consistent with previous research, the present study found that the TD group outperformed the DLD group significantly in the field of receptive language, cognitive ToM (e.g., Nilsson and de López 2016), and socio‐emotional functioning (e.g., Durkin and Conti‐Ramsden 2007). Thus, this current study indicates that the problems in these three areas, already found in school‐aged children with DLD, persist in adolescence. In addition, receptive language was associated with cognitive shifting (EF), cognitive ToM, affective ToM, and four aspects of social‐emotional functioning (i.e., task approach, coping with difficulties, adapting, and collaboration). This is consistent with previous research highlighting the crucial role of language in EF, ToM, and social‐emotional functioning (Camminga et al. 2021). Furthermore, cognitive ToM was found to be a significant predictor of socio‐emotional functioning. Specifically, parents of adolescents with lower levels of cognitive ToM reported more difficulties in the socio‐emotional functioning of their adolescents. This finding aligns with neuropsychological insights, indicating that problems in socio‐emotional functioning can be cognitively explained in terms of ToM (Vissers and Koolen 2016). The finding that socio‐emotional functioning was best explained by cognitive ToM may also reflect the developmental stage of the participants. During adolescence, higher‐order cognitive ToM abilities, such as understanding complex intentions and perspectives, are still maturing, while affective ToM typically stabilizes earlier. Consequently, cognitive ToM may play a particularly salient role in explaining socio‐emotional functioning at this age.

In contrast with earlier studies in younger children with DLD, we did not find significant differences between the DLD and TD group on EF. In addition, we found no significant correlations between EF and socio‐emotional functioning in adolescents with and without DLD. This absence of an association is an important finding, suggesting that EF may play a less prominent role in socio‐emotional functioning during adolescence than previously assumed. The lack of support for hypotheses regarding EF can be explained by several possible factors. First, executive function remains a confusing construct due to the various definitions, diverse theories, conceptualizations, and the many different measures associated with it in the literature (Anderson 2002; Fisk and Sharp 2004; Huizinga et al. 2006; Miyake et al. 2000). This makes it challenging to establish content validity. Consequently, using different instruments to measure EF can produce completely different results, complicating the comparison of EF research between studies. A second reason may be that working memory in this study was measured with language‐independent tasks (i.e., the task was primarily visual, thus not relying on complex language skills). Although a meta‐analysis of children with DLD found deficits in visual working memory compared to TD peers, it is suggested that the deficit in verbal working memory is two to three times larger than that in visual working memory (Vugs 2013). The lack of differences found between adolescents with and without DLD in visual working memory does, therefore, not provide evidence for a similar absence of an effect on verbal working memory. Further research could clarify this. A third reason could be related to the way EF was measured. In this current study, we used ‘cool’ EF tasks (i.e., a single task involving neutral stimuli, aimed at measuring a specific skill under calm and controlled testing conditions) to measure EF. However, the so called ‘hot’ EF in daily social life may be characterized by more complex interacting skills and functions (Hobson et al. 2011). Based on this information, it could be that adolescents with DLD do not perform worse on tasks that measure ‘cool’ EF as an isolated skill but may still struggle with ‘hot’ EF in daily life. Future research should aim to compare these two assessment methods in order to clarify the relationship between performance on behavioural measurements in research settings and in real‐life situations. A fourth explanation for the absence of significant results may be that the current study did not cover the full spectrum of executive functions. In addition to working memory and cognitive flexibility, other EF components such as attention and inhibitory control are essential for regulating emotions, maintaining focus on relevant social cues, and adapting behaviour to changing social situations. Recent neuropsychological perspectives highlight that language, EF, and socio‐emotional development are deeply interconnected rather than independent processes (Vissers and Tomas 2019). Within this framework, difficulties in attention or inhibitory control may indirectly contribute to socio‐emotional challenges by limiting an individual's ability to process, interpret, and respond effectively to social information. Future research should therefore aim to include these EF components to provide a more comprehensive understanding of how EF relates to socio‐emotional adjustment in adolescents with and without DLD.

Unexpectedly, we found that language is not a significant predictor of socio‐emotional functioning in adolescents with and without DLD. Moreover, we found no significant relationships between receptive language and four categories of social‐emotional functioning (i.e., standing up for oneself, self‐knowledge, feelings, and presenting). This unexpected finding may be due to the fact that only receptive language skills were measured in this study. In the literature, a clear distinction is made between receptive and expressive language skills (Leonard 2009), each with its own consequences for social‐emotional functioning. Previous research, for example, has shown that children with receptive language disorders are at greater risk of developing problems with social‐emotional functioning than children who present only with expressive language disorders (Toppelberg and Shapiro 2000). However, children with expressive language difficulties appear to exhibit more shyness, combined with a fear of expressing themselves (Caulfield et al. 1989). Building on this literature, we expect that there may be a significant correlation between expressive language skills and the other (currently not significant) categories of social‐emotional functioning. In addition, we expect that a more comprehensive language assessment, including both receptive and expressive skills, may be predictive of social‐emotional functioning. Future research is needed to further investigate and confirm these findings.

Contrary to expectations, this study shows that there was no significant correlation between emotion recognition and socio‐emotional functioning. This finding is not in line with a recent meta‐analysis in TD adolescents (Zhang et al. 2023), who found that weaker emotion recognition skills were associated with higher internalizing behavioural problems. However, a difference between this current study and the study by Zhang et al. (2023) is that Zhang's meta‐analysis examined correlations between emotion recognition and internalizing problems with clinical significance (e.g., depression/anxiety). In contrast, our study focused on the relationship between emotion recognition and more everyday socio‐emotional issues (such as making connections, discussing emotions, etc.). The results of our study are, however, in line with research in a more clinical sample of adolescents with ADHD (Fitzpatrick et al. 2018), who also found no relation between affective ToM and social behaviour. These researchers suggested that the absence of the expected correlation may be related to the static nature of the emotion recognition task, making this task possibly unsuitable for discovering more subtle relationships. Given that emotion recognition in this study is measured similarly to the approach used by Fitzpatrick et al. (2018) (i.e., estimating emotions based on facial photographs), it is possible that our task may also lack sensitivity for detecting differences. It is interesting to explore through future research whether there is a correlation between emotion recognition and more internalizing problem behaviour. Furthermore, it is advisable to assess emotion recognition using a more dynamic approach rather than relying on static measures.

Finally, this study demonstrated significant individual variability in language skills, EF, ToM, and socio‐emotional functioning within the DLD group. This large variance suggests that the group of adolescents with DLD is not homogeneous and that factors such as the severity of the language disorder may be a consequence of this variability. These findings are consistent with previous studies that have also identified significant variability among individuals with DLD (for EF, see Henry et al. 2012; for ToM, see Farrant et al. 2006). Given this variability, it is essential that interventions for individuals with DLD are tailored to their specific cognitive profiles in order to be effective. Further studies with a larger sample of adolescents with DLD could further investigate these topics.

### 4.1 Study Limitations

Several limitations of this study should be acknowledged. First, these findings are preliminary since this is one of the first studies to examine socio‐emotional functioning and underlying constructs in adolescents with DLD. Future research is needed utilizing larger sample sizes with a wider range of social, cognitive (EF, ToM) and linguistic abilities to more fully understand the problems in DLD and the relationships among them. A second limitation is that in this study, only the parents’ perspective was used to assess socio‐emotional functioning. Since having an own identity and peer relations become more important during adolescence, it would be interesting to include peer‐ and self‐assessments as well. A third limitation of this study is the absence of a test for expressive language skills. Current literature clearly distinguishes between the effects of receptive and expressive language problems on social‐emotional functioning (Toppelberg and Shapiro 2000; Caulfield et al. 1989). Since this study relies solely on a measure of receptive language, it is possible that we may not have identified difficulties in social‐emotional functioning that stem from expressive language issues. Fourth, the cross‐sectional design of the study prevents us from drawing conclusions about the causation and malleability of the constructs under study. Experimental intervention studies may help to clarify whether training in EF and ToM may actually contribute to promoting socio‐emotional functioning in adolescents with and without DLD.

## 5 Conclusions and Clinical Implications

This study showed that significant differences in language, cognitive ToM, and social‐emotional functioning exist between adolescents with DLD and TD. Specifically, cognitive ToM was found to be associated with lower socio‐emotional functioning in these youths. These findings suggest that the nature of DLD requires considering social and emotional behaviour. Since difficulties in socio‐emotional functioning are associated with negative implications later in life, it is important to study whether adequate, targeted and effective treatment methods can be developed to improve socio‐emotional functioning. In attendance of such studies, schools and care may tentatively be advised not only to address the primary language problems but also to provide specific training aimed at improving socio‐emotional functioning. Since the differences in socio‐emotional functioning seem to be largely explained by differences in cognitive ToM, it is suggested to specifically address this cognitive ability and to examine whether training X improves Y. Based on the present results, interventions for adolescents with DLD should prioritize strengthening cognitive ToM skills, such as perspective‐taking and understanding others’ thoughts, feelings, and intentions. Evidence shows that engaging in mental state conversations—structured dialogues about what people think, feel, or want—can effectively promote ToM development (Ruffman et al. 2002; Durrleman 2020). Moreover, fostering self–other distinction, or reflecting on how one's own thoughts and emotions differ from those of others, may enhance social understanding (Santiesteban et al. 2012). However, recent research shows that ToM interventions often improve performance on experimental ToM tasks but do not generalize to everyday life, possibly because these interventions do not sufficiently focus on applying ToM skills in real‐world social contexts (Smit et al. 2024; Dyrda et al. 2020). Emerging approaches such as virtual reality (VR)‐based interventions may therefore offer promising opportunities to practice both cognitive and affective ToM within realistic and interactive social environments (Arts et al. 2025).

Taken together, these insights emphasize that supporting adolescents with DLD requires more than addressing language problems alone; it also involves helping them develop the socio‐cognitive tools needed to navigate complex social worlds. Given the complexity of their needs, a multidisciplinary approach seems essential, especially as they enter the period of adolescence.

## Funding

This study was supported by funding from National Regieorgaan Onderwijsonderzoek (grant number 4O.5.19630.066).

## Conflicts of Interest

The authors declare no conflicts of interest.

## Data Availability

All research data and codes relevant to the results described in this article are available in the Radboud Data Repository via: https://doi.org/10.34973/g7sy‐yc44
